# An Insight into Practices Associated with the Control of Internal Parasites in the Dairy Goat Herds of Romania: A Questionnaire Survey

**DOI:** 10.3390/ani14162375

**Published:** 2024-08-16

**Authors:** Adrian-Valentin Potârniche, Constantin Cerbu, Diana Olah, Emilia Trif, Gianluca D’Amico, Adriana Györke, Marcin Mickiewicz, Zofia Nowek, Michał Czopowicz, Dorina Nadolu, Andreea Hortanse Anghel, Jarosław Kaba

**Affiliations:** 1 Department of Infectious Diseases, Faculty of Veterinary Medicine, University of Agricultural Sciences and Veterinary Medicine of Cluj-Napoca, Mănăștur Str. 3–5, 400372 Cluj, Romania; adrian.potarniche@usamvcluj.ro (A.-V.P.);; 2 Department of Parasitology and Parasitic Diseases, Faculty of Veterinary Medicine, University of Agricultural Sciences and Veterinary Medicine of Cluj-Napoca, Mănăștur Str. 3–5, 400372 Cluj, Romania; 3 Division of Veterinary Epidemiology and Economics, Institute of Veterinary Medicine, Warsaw University of Life Sciences—SGGW, Nowoursynowska Str. 159c, 02-776 Warsaw, Poland; 4 Institute of Research and Development for Sheep and Goat Breeding Palas Constanța, 248 I.C. Brătianu, 900316 Constanța, Romania; 5 Department of Natural Sciences, Faculty of Natural Sciences and Agricultural Sciences, Ovidius University of Constanța, Universității Str. 1, 900470 Constanța, Romania

**Keywords:** anthelmintic resistance, benzimidazoles, gastrointestinal nematodes, levamisole, macrocyclic lactones, small ruminants

## Abstract

**Simple Summary:**

Parasitic infections, especially those caused by a particular group of roundworms called gastrointestinal nematodes, are one of the most important diseases of goats worldwide. The widespread and uncontrolled use of deworming drugs (anthelmintics) makes nematodes increasingly resistant, and this phenomenon is called anthelmintic resistance. Romania has one of the highest goat populations in Europe, but little is known about how widespread and resistant gastrointestinal parasites are in this country. That is why we decided to carry out this survey—to find out how Romanian farmers fight parasites in their goats and what their opinion is on the presence of parasites and the effectiveness of deworming drugs in their herds. Our findings are disappointing. Most farmers deworm goats when they think it is necessary, not when laboratory tests show that they really need it. Moreover, they use deworming drugs too often and at too low doses. Such practices are very likely to stimulate parasites to develop anthelmintic resistance, and only intensive education programs for farmers and veterinarians can stop this process.

**Abstract:**

The widespread and uncontrolled use of anthelmintic products has contributed to the emergence of anthelmintic resistance (AR). This phenomenon globally threatens the productivity and welfare of small ruminants. A questionnaire consisting of 34 questions was handed to 234 goat farmers across Romania to gain insight into control practices against internal parasites and the farmers’ perception of the parasitic infections present in their herds and the efficacy of anthelmintic treatments. The majority of farmers (88.5%) admitted they had never submitted fecal samples for parasitological laboratory analysis, and 77.4% had treated the animals on their own. In general, the farmers dewormed their goats based on visual body weight estimation. Prophylactic anthelmintic treatment was practiced by more than 85% of the farmers. A traditional control approach based on treating the entire herd at fixed time intervals is widespread among Romanian goat and sheep farmers. The most commonly used anthelmintic drugs in the previous 3 years (2021–2023) were benzimidazoles (85.5%) and macrocyclic lactones (81.6%). Poor anthelmintic efficacy was suspected by 14.5% of farmers, and the minority (18.0%) considered internal parasites as a problem in their herds. Regarding the farmers’ perception of the presence of parasites, there was a significant level of uncertainty. This is the first survey carried out in Romanian goat herds, and it provides up-to-date information on practices aimed at controlling internal parasites.

## 1. Introduction

Gastrointestinal nematode (GIN) infections lead to clinical and subclinical diseases, which result in considerable economic losses, affecting the profitability and sustainability of goat farming worldwide [1,2]. Anthelmintic treatment remains the primary method for managing GIN infections in ruminants, relying on three main classes of chemical compounds: benzimidazoles (BZs), macrocyclic lactones (MLs), and imidazothiazoles, notably levamisole (LEV) [3]. As a result, the common and uncontrolled use of these drugs has contributed to the emergence of anthelmintic resistance (AR) [4,5,6]. This phenomenon precludes the effective control of parasitic diseases, threatening small ruminants’ productivity and welfare globally [7,8]. AR is increasingly recognized as a widespread challenge in many regions and European countries [9,10,11,12,13], including Romania [14].

AR can only be detected using certain diagnostic tests. The fecal egg count reduction test (FECRT) is an in vivo test (i.e., test carried out directly on animals) recommended by the World Association for the Advancement of Veterinary Parasitology (WAAVP) for diagnosing and monitoring AR [15,16]. FECRT is considered highly accurate and reliable, but it is also time-consuming and laborious because the effectiveness of each anthelmintic has to be separately tested on a group of animals from the herd. Therefore, in field studies, it is often substituted by in vitro tests such as the egg hatch test (EHT) and larval development test (LDL), which only require collection of a single fecal sample and its delivery to the laboratory in proper conditions. On the other hand, the laboratory has to show expertise in parasitological techniques such as larval culture, which is not common. These factors make the diagnostics of AR hardly available in many regions of the world, and as a result AR remains a vastly underrated veterinary problem.

Several key factors contribute to the development of AR in parasites. These include frequent use of anthelmintics, underdosing, continuous use of the same class of anthelmintics over extended periods, and certain farm management practices [12,17,18,19,20,21,22]. These factors are particularly important in goats, which require higher doses for effective treatment due to faster metabolism and drug elimination than cattle or sheep [23,24,25]. Additional risk factors include the dose-and-move strategy [21], targeting and timing of mass treatment [26], and the introduction of animals harboring resistant parasites into herds [27].

AR can develop against a single class or multiple classes of anthelmintics, and the latter phenomenon is known as multidrug resistance (MDR). AR appears to correlate with the frequency of use of different anthelmintic classes in veterinary practice [9,28]. Due to the increased reporting of AR, efforts have shifted towards sustainable parasite control methods to slow its progression. A fundamental aspect of implementing sustainable parasite control involves relying on routine laboratory diagnostics prior to making therapeutic decisions [29,30].

In Romania, antiparasitic products must be prescribed by veterinarians. However, to date, there is no systemic surveillance program for their use. BZs and MLs are the only anthelmintic classes licensed for goats. Of these, BZs are most commonly used, mainly due to their relatively short withdrawal period [14]. In Romania, only a single dairy goat herd with AR in GINs has so far been described, and *Haemonchus contortus* turned out to be the only GIN resistant to both BZs and MLs [14]. According to EUROSTAT [31], Romania has Europe’s third-largest goat population, after Greece and Spain. Thus, it is of paramount importance to gain insights into Romanian goat herds’ control practices against internal parasites and understand how these practices can affect the further development of AR.

Questionnaire surveys on parasite management practices in small ruminants have previously been conducted in Belgium [32], Canada [33], Denmark [34], Ethiopia [35], France [36], Italy [37], the Netherlands [38], Norway [19,39], the Slovak Republic [40], Spain [41], and Thailand [42]. However, no similar survey has been carried out in Romania.

The present study aimed to gather information about the practices associated with the control of internal parasites in dairy goat herds in Romania and to learn farmers’ subjective perception of the presence of internal parasites and the effectiveness of anthelmintic treatments in their herds.

## 2. Materials and Methods

### 2.1. Questionnaire Development and Distribution

A questionnaire survey based on in-person interviews was carried out between September 2023 and May 2024. The study population included goat herds registered in the National Association of Goat Breeders in Romania (CAPRIROM). The CAPRIROM gathers 1432 goat farms. A pilot survey was conducted on a small group of goat farmers to ensure that the questionnaire was sufficiently transparent and comprehensible to avoid misinterpretation. The minimum sample size was calculated separately for each macro-region of Romania, assuming the expected prevalence of an investigated phenomenon of 50%, accepted error (precision) of ±15%, and level of confidence of 95%. The minimum required sample size was 43 farms from each macro-region based on the Nomenclature of Territorial Units for Statistics (NUTS). The farms were randomly selected in each macro-region [43].

Before the official onset of the survey, the farmers selected to participate were informed via the CAPRIROM about the objectives and the potential impact that the results could have on the control of parasitic infections. To ensure the most accurate responses, the printed questionnaires were provided to the respondents by local veterinarians collaborating with the CAPRIROM and the farmers were asked to complete the questionnaires in their presence so that the veterinarians could assist them and then immediately collect the questionnaires.

The questionnaire was prepared in Romanian and consisted of 34 questions (Supplementary Materials). It was developed based on the questionnaire used in the study of Gravdal et al. [19]. The questionnaire was split into four parts: the first part included general information about the farms (10 questions), the second part contained information regarding parasite control practices (14 questions), and the third (4 questions) and the fourth (6 questions) part focused on anthelmintic treatment and farmers’ perception of parasitic infections in their herds, respectively. The questionnaire did not contain any personal or contact details of respondents, except for the farm’s ID number registered in the CAPRIROM.

### 2.2. Data Processing and Statistical Analysis

The normal distribution of numerical data (herd size) was evaluated by normal probability Q-Q plots and the Shapiro–Wilk W test. As the normality assumption was rejected, numerical variables were presented as median, interquartile range (IQR), and range, and compared between groups using the Mann–Whitney U test (2 groups) or Kruskal Wallis H test with Dunn’s post hoc test (>2 groups). The correlations between numerical variables were determined using Spearman’s rank correlation coefficient (R_s_). Categorical variables were expressed as counts and proportions. The 95% confidence intervals (CI 95%) for proportions were calculated using the Wilson score method [44]. Categorical variables were compared between groups using the maximum likelihood G test or Fisher’s exact test (if the mean expected frequency per cell of the contingency table was <6) [45]. A significance level (α) was set at 0.05. All statistical tests were two-tailed. The statistical analysis was performed using TIBCO Statistica 13.3 (TIBCO Software Inc., Palo Alto, CA, USA).

## 3. Results

**Figure 1:**
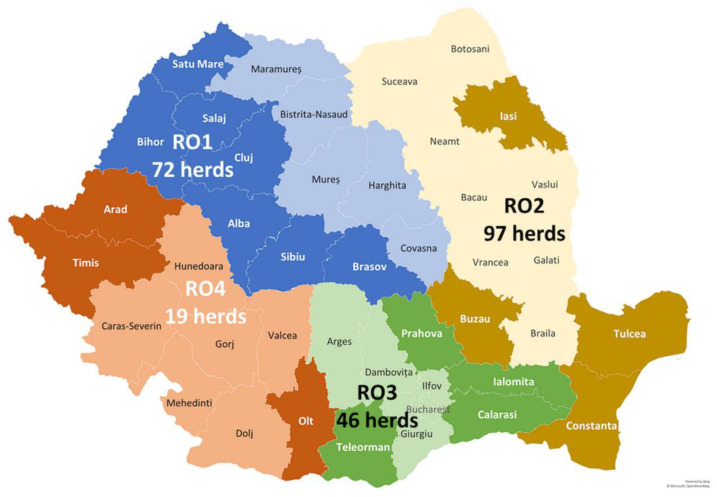
Four NUTS macro-regions (RO1–RO4) and 18 counties of Romania in which the herds included in the study were located (counties marked with darker color and white names).

The study was carried out on 234 farms located in 18 counties from 4 macro-regions (RO1–RO4) of Romania, with the highest proportion of farms from RO2 (97/234, 41.5%), followed by RO1 (72/234, 30.5%) and RO3 (46/234, 19.6%), and the lowest in RO4 (19/234, 8.1%) (Figure 1). The number of farms in the RO4 macro-region was lower than required to ensure representativeness.

### 3.1. General Information about the Holdings and Pastures

**Figure 2:**
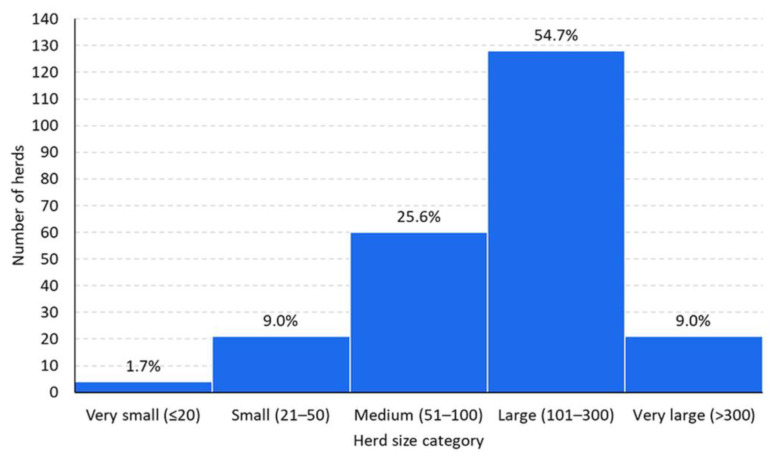
Frequency distribution of goat herds according to their size.

**Figure 3:**
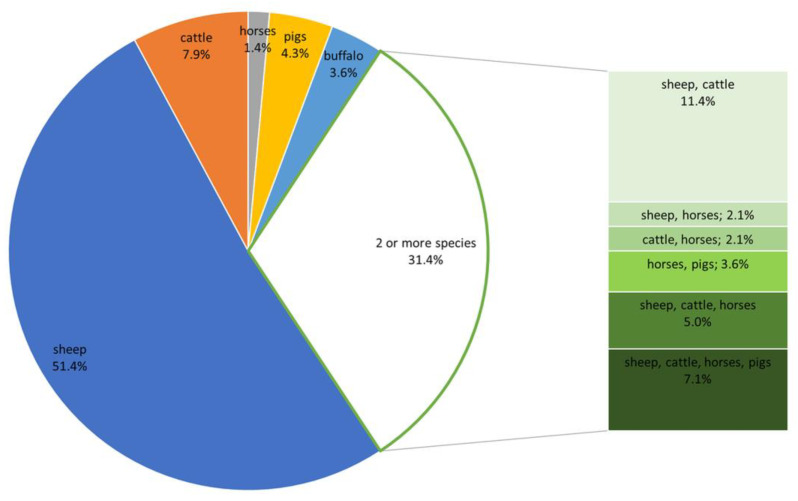
The distribution of herds according to other livestock species kept together with goats.

Herds counted from 12 to 718 adult goats, with a median (IQR) of 134 (80–220) adult goats (i.e., goats aged >1 year). These herds kept 37,934 goats in total and accounted for approximately 20% of the total number of goats registered in the CAPRIROM and 3.5% of the total goat population of Romania. Following the commonly used classification of goat herds [46,47], the herds were categorized as very small (<20 animals), small (21–50), medium (51–100), large (101–300), and very large (>300). Over half of the herds enrolled in the study were large (128/234, 54.7%), whereas small herds were the least common (4/234, 1.7%) (Figure 2). The largest goat herds were located in RO2 and the smallest in RO1, and the difference between the size of herds of these two macro-regions was significant (*p* < 0.001). The most popular breed was the native Carpathian (kept in 191/234, 81.6%), followed by French Alpine (18/234, 7.7%), Anglo-Nubian (17/234, 7.3%), Saanen (5/234, 2.1%), and Banat White (3/234, 1.3%). The animals in all herds were housed on solid floors covered with straw bedding. In almost 60% of the herds (140/234, 59.8%), the goats were kept together with other livestock species, of which sheep were the most common (108/140, 77.1%). A single livestock species was reported in 68.6% (96/140) of the herds, and 2 and 4 livestock species in the remaining 31.4% (44/140) herds (Figure 3). An intensive farming system (defined as no grazing on pasture) was practiced in only 7.7% (18/234) of the herds, raising exclusively non-traditional goat breeds such as French Alpine or Anglo-Nubian (*p* < 0.001). The herds with intensive farming system were uniformly distributed over 4 macro-regions (*p* = 0.324). The herds practicing intensive farming were significantly smaller than the extensively farmed herds (*p* = 0.001).

**Figure 4:**
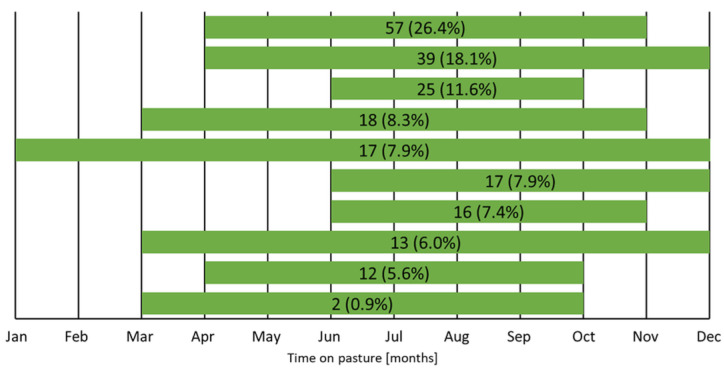
Seasonal grazing patterns (part of a year in which goats grazed on the pasture) in goat herds. Numbers and proportions of herds from 216 herds with an extensive farming system are presented on bars.

**Figure 5:**
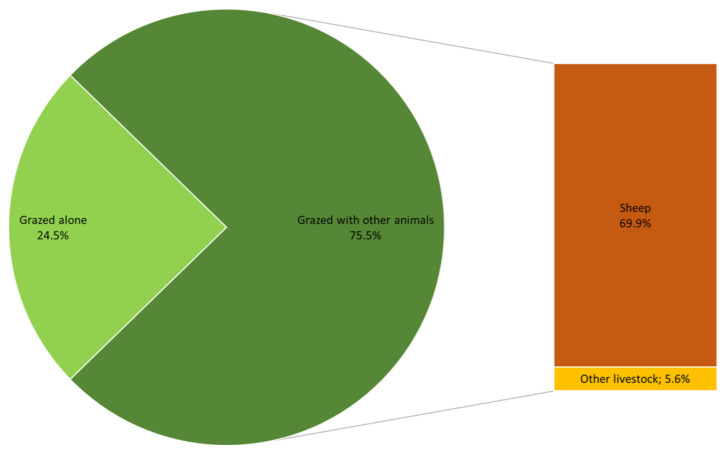
Grazing practices in 216 Romanian goat herds with an extensive farming system.

April was the month when goats were most often turned out onto pasture (108/216, 50.0%), whereas the most common month of returning to the stable was November (91/216, 42.0%). In the majority of herds (199/216, 92.1%), goats spent between 4 and 9 months on pasture, with a median (IQR) of 7 (6–8) months. The remaining herds (17/216, 7.9%) were kept on the pasture for the entire year (Figure 4). The goats grazed alone in only 24.5% (53/216) of herds, while in the rest they grazed together with other livestock animals, mainly with sheep (Figure 5). Regular pastures were the primary grazing areas (170/216, 78.7%), followed by forests (38/216, 17.6%). When on pasture, goats in most herds (149/216, 68.9%) drank surface water.

### 3.2. Parasite Control Practices

Most farmers were satisfied with the advice received from local veterinarians on parasite control practices (163/234, 69.6%). In total, 56.4% (132/234) of the farmers were in regular contact with their local veterinarians regarding parasite control. However, 33.3% (78/234) of them never discuss with a veterinarian before starting an antiparasitic treatment. The veterinarian was viewed as the primary advisor for antiparasitic treatment by 62.0% (145/234) of farmers, followed by the “online advisor” (internet) by 13.2% (31/234) farmers. The previous experience of the farmers (165/234, 70.5%) was the primary factor in determining when to treat the goats against internal parasites. The emergence of clinical symptoms in goats (27/234, 11.5%) was another deciding factor, while only 3.4% (8/234) of the participants used parasitological laboratory analysis (fecal egg count, FEC) as a basis for treatment decision. The main factors taken into account by farmers when choosing the antiparasitic treatment were their subjective opinion on drugs’ effectiveness (109/234, 46.6%) and the withdrawal period (63/234, 26.9%). The majority of farmers (207/234, 88.5%) admitted that they had never submitted fecal samples for parasitological laboratory analysis. The deworming of animals was performed directly by farmers (without any assistance from a veterinarian or technician) in 77.4% (181/234) of the herds, and the main source of purchasing antiparasitic drugs was the veterinary pharmacy (201/234, 85.9%). Quarantine of newly acquired animals was not implemented by half of the farmers (115/234, 49.1%), but most farmers dewormed newly purchased animals before introducing them into the herd (150/234, 64.1%). The determination of anthelmintic dosage was based mainly on visual assessment of the body weight of animals (191/234, 81.6%), and only 2.6% (6/234) farmers weighed individual goats (Table 1). Only 21.8% (51/234) of the farmers knew that goats required higher doses of most anthelmintics compared to sheep. Most farmers used syringes (158/234, 67.5%), followed by drenching guns (71/234, 30.4%), as a tool for oral administration of anthelmintics.

### 3.3. Anthelmintic Treatment

Only three farmers (1.3%) declared never to have dewormed their goats. Over 60% of the farmers (149/234, 63.7%) dewormed their goats two or three times a year, and a quarter of them (62/234, 26.5%) dewormed more often (>3 times/year). The most commonly used anthelmintics in the previous 3 years (2021–2023) were BZs (200/234, 85.5%) and MLs (191/234, 81.5%), followed by the rarely used LEV (14/234, 6.0%) (Table 2). Albendazole and ivermectin were the main representatives of their classes.

Most farmers reported using two classes of anthelmintics (148/234, 63.2%), while 30.8% (72/234) used drugs from the same class, and 5.6% (13/234) from three different classes. The vast majority of farmers (202/234, 86.3%) dewormed their animals as a preventive measure. Of these, 55.5% (112/202) had not encountered any previous issues with internal parasites, while 44.5% (90/202) had dealt with such problems before. Only 13.7% (32/234) treated in response to specific symptoms.

### 3.4. Farmers’ Perception of Parasitic Infections

**Figure 6:**
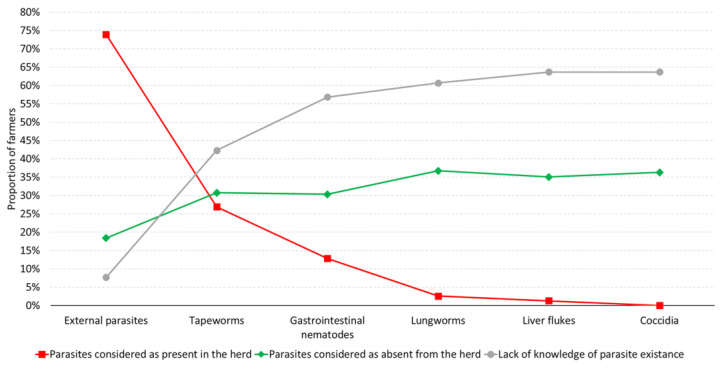
Farmers’ opinion on the occurrence of particular groups of parasites in their herds.

Regarding the farmers’ perception of the presence of internal parasites, there was a significant level of uncertainty, with 42.3% to 63.7% of farmers unsure about the parasite species that was present in their herds. The lack of awareness was very strongly negatively correlated with the suspected frequency of occurrence of parasites (R_s_ = −0.99, *p* < 0.001)—the less the farmers were aware of the existence of a given type of parasites, the less often they declared these parasites were present (Figure 6). The farmers were more aware of external parasites (173/234, 73.9%) and tapeworms (63/234, 26.9%) than of other parasites. In their herds, the farmers observed cough (109/234, 46.6%) and diarrhea (103/224, 44.0%) as the most predominant clinical signs, whereas anemia (69/234, 29.5%) and sudden death (67/234, 28.6%) were least frequently noted (Table 3).

Less than half of the farmers (96/234, 41.0%) knew that the rotation of pasture could reduce parasitic infections, but only a quarter (57/234, 24.4%) would have had the possibility to rotate the pasture from one year to another. The majority of the farmers (200/234, 85.5%) regarded deworming as effective. A minority (18.0%, 42/234) considered internal parasites an important problem in their herds (Table 4).

## 4. Discussion

This is the first survey conducted in Romania aimed at providing information on control practices against internal parasites (particularly GINs) in Romanian goat herds and on farmers’ awareness of these parasites and the effectiveness of antiparasitic treatments.

### 4.1. Parasite Control Practices

Our study indicates that more than half of the farmers maintain regular contact with their veterinarians throughout the year regarding parasite control. However, one-third of farmers initiate antiparasitic treatments without prior veterinary consultation. This practice raises the risk of improper use of anthelmintics, potentially exacerbating issues related to AR [10]. Unfortunately, avoiding professional advice before treatment initiation is a common practice among sheep and goat farmers in Romania [14].

Farmers tend to rely on scheduled treatments based on previous experiences, housing times, or pasture rotation rather than opting for parasitological laboratory analysis. This trend has been observed in various studies across different geographic regions [19,32,35,38,42,48]. In our survey, the majority of farmers (88.5%) admitted that they had never submitted fecal samples for analysis, highlighting a significant gap in the implementation of diagnostic-driven treatment approaches. This raises great concern compared to practices in other regions, where regular FEC monitoring is more common [38]. For instance, a study from the UK showed that a higher proportion of farmers employed FEC as part of their parasite management strategy, acknowledging its role in preventing AR by enabling targeted treatments [49]. The same situation was reported in New Zealand, where the adoption of FEC and other diagnostic tools was higher due to extensive farmers’ education programs emphasizing the importance of such practices in managing AR [50].

Furthermore, most farmers deworm animals by themselves, without any veterinary or technical assistance. Similar situations have been noted in surveys in other countries, such as Ethiopia, Norway, Poland, Thailand, and the UK [19,28,35,42,48]. This can be explained by several reasons. Farmers might undervalue diagnostic testing if they achieve satisfactory results with their current deworming strategies [39]. They may also view diagnostic testing as an unnecessary expense, especially if FEC results do not clearly indicate further action, for example, due to the lack of the threshold value of eggs per gram (EPG), which suggests the need for anthelmintic treatment. For many Romanian farmers, the financial aspect is the one that prevails. Farming involves numerous challenges, such as time management, stock health and welfare, maintenance, regulatory compliance, and consumer demands [51]. Given these priorities, parasitological testing often takes a backseat, particularly when anthelmintic treatment efficacy is not perceived as a problematic issue [19]. In Romania, anthelmintics must be prescribed by veterinarians; however, many farmers can still procure these products at their own discretion. Unfortunately, these practices will persist without a strict surveillance program for the misuse of such products. Another potential factor could be the lack of encouragement from veterinarians regarding parasitological diagnostics. In a questionnaire survey, over one-third of Romanian veterinarians who regularly worked with small ruminants did not perform parasitological laboratory analysis before treatment, referring to farmers’ unwillingness to pay for the diagnostic tests [52].

Incorrect administration and dosing of anthelmintics considerably contributes to the development of AR. Visual body weight assessment is frequently employed to determine anthelmintic doses in veterinary medicine, but it often leads to underdosing. This enables the survival of heterozygous resistant worms, thereby promoting the selection of resistant strains [20]. In our study, more than 80% of the farmers calculated the dose of anthelmintics based on visual assessment of the animals’ body weight. A similar situation was observed in Norway [53] and Denmark [34], while in Canada only less than half of the farmers estimated the body weight in this manner [33].

Underdosing is not only the consequence of underestimation of goat’s body weight but may also be caused by applying too low a dose per 1 kg of body weight. Less than a quarter of the farmers knew that the dose of anthelmintics in goats differed from sheep or cattle. This situation is understandable given that this information is not mentioned in the leaflet of any anthelmintic licensed for goats in Romania. Most farmers used medical syringes to drench their goats, which are less prone to dosage errors compared to the drenching guns that require frequent calibration to maintain optimal precision of dosing [33].

Implementing quarantine routines is essential for a sustainable parasite control program, as it helps prevent the introduction and spread of parasites, including drug-resistant strains. Data from this study show that farmers are more used to deworming animals immediately after the purchase (64%) than to placing them on quarantine (49%). Gravdal et al. reported almost identical results in Norwegian sheep [19] and roughly 54% of farmers from the UK never performed quarantine when they purchased sheep [48]. Without proper quarantine and treatment, new resistant parasites can be introduced into herds, further exacerbating the existing problems.

### 4.2. Anthelmintic Treatment

The goats were mostly dewormed two or three times a year. More than a quarter of the farmers (26.5%) treated them even more often. The situation is alarming if we compare these results with recent studies from other countries. In Slovakia, the sheep farmers dewormed their animals on average 1.4 times per year [40]; in Norway [19], 1.5 times [40]; while in Poland, the majority of goat farmers dewormed 2 times a year [17]. However, the situation is better than the one described in Thailand, where goat farmers dewormed their animals 5 times a year on average [42]. An increasing frequency of administration of an anthelmintic is a well-known factor accelerating the development of AR [8,20,54,55].

In Romania, during the last 3 years, the most commonly used anthelmintics were BZs (mainly albendazole) and MLs (mainly ivermectin). In many cases, both classes were used in the same year. These results are consistent with most studies conducted so far, with minor differences between the two mentioned classes of anthelmintics [17,19,36,40,48]. BZs are cost-effective and have a short withdrawal period, which is crucial for farmers who prioritize obtaining dairy products from their herds [14]. As previously mentioned, Romanian farmers pay the most attention to the short withdrawal period and subjective efficacy when selecting an anthelmintic drug. MLs (especially ivermectin) are very commonly used anthelmintics, despite the extended withdrawal time for milk. However, we know from our experience that many farmers use MLs before housing, when the goats are already in the dry-off period. Unfortunately, the seasonality of these treatments was not investigated in this study. Additionally, many Romanian farmers use ivermectin because it is also effective against external parasites. On the other hand, LEV is seldom used compared to other European countries, where its frequent use has led to the development and spread of AR to LEV [22,28,56]. The most likely reason is the lack of licensed LEV products for goats in the Romanian market. These products are licensed only for sheep and cattle, and many goat farmers do not know about the possibility of using such products in goats. The distribution of AR appears to correlate with the popularity of anthelmintic classes used in veterinary practice [5,28]. The only study published so far in Romania revealed increased resistance to both BZs and MLs but not to LEV in a dairy goat herd [14].

Prophylactic anthelmintic treatment was widely practiced by more than 85% of Romanian farmers. Traditional control programs against internal parasites based on the use of broad-spectrum anthelmintic drugs in the entire herd at fixed temporal intervals are widely spread among goat and sheep farmers. This approach only worsens the already unfavorable situation regarding AR. There is a need for rapid implementation of education programs for farmers and veterinarians about currently recommended strategies that can limit or slow down the progress of AR. One of these strategies is “smart deworming”, also known as targeted selective treatment (TST), in which only the animals “in need” are treated [38]. One study indicated that the adoption of informed treatment practices is significantly influenced by farmers’ attitudes as well [57]. Implementing TST requires much closer collaboration between the farmer and the veterinarian, with understanding and willingness on both sides. Initially, this process generates substantial financial and temporal costs. However, eventual benefits for the farmers are significant and attractive: fewer animals to be treated, reduced time and effort spent on deworming, reduced drug use, saving more money, and maintaining the effectiveness of available anthelmintics, resulting in healthier animals [29,58].

### 4.3. Farmers’ Perception of Parasitic Infections

A significant level of uncertainty was evident in how farmers perceived the presence of parasites. The more often farmers were unaware of the existence of a given parasite, the less often they claimed their goats had it. They were more conscious of the presence of tapeworms, likely because of the visible proglottids in the feces. Data regarding the epidemiology of GINs in goats are scarce in Romania. Potârniche et al. [14] reported the presence of *Teladorsagia circumcincta*, *H. contortus*, and *Oesophagostomum* spp. in a dairy goat herd from the northwestern part of the country. However, the distribution of these parasites in the country is unknown. In another study, PCR analysis showed that three out of four herds from Transylvania were positive for *Haemonchus placei*, *T. circumcincta*, *Trichostrongylus colubriformis*, and *Oesophagostomum venulosum*, while *H. contortus* was identified in the last herd (manuscript in preparation).

As already mentioned before, most farmers treat their goats very often using accidentally chosen products without knowing the “enemy” they are dealing with. Low infection awareness has been considered an important obstacle to the implementation of sustainable parasite control [19,59]. Over half of the respondents were unaware that pasture rotation could reduce parasitic infection. Even among those who were aware, the vast majority lacked the means to implement this measure. This underscores the importance of following “modern” strategies such as the TST approach to maintaining refugia levels in all pastures. In general, the farmers considered the anthelmintic treatment effective, similar to reports from Ethiopia, France, Norway, Thailand, and the UK [19,35,36,42,48]. The herds in which parasites were viewed as an important problem were significantly smaller, practiced mainly in extensive systems of farming, grazed in backyards, and were located primarily in RO1. This is directly linked to the frequency of treatment. The farmers tend to increase the frequency of deworming when they are not satisfied with previous results.

Interestingly, the owners of these herds used the internet more often as a source of knowledge and observed clinical symptoms in their goats (with the exception of diarrhea) much more often. Perhaps, many of them attribute the lack of treatment efficacy to veterinarians and seek information from alternative sources. Farmers with small herds are more likely to notice the development of clinical signs and the lack of efficacy of anthelmintic treatments compared to those with larger herds. Small herds are typically family-run and directly involve the owner, while larger herds often employ a shepherd to care for the animals, who is not always attentive to these aspects. Extensive farming is much more common in small ruminants in Romania than intensive farming, and goats are prone to parasitic infections with GINs, especially when they graze in backyards where the reinfections are permanent.

## 5. Conclusions

The present study reveals that the anthelmintic treatment routines practiced in Romanian goat herds may significantly contribute to the development of AR. The vast majority of farmers administer treatments themselves, for prophylactic purposes, doing so very frequently and using inappropriate doses. Implementing an education program for farmers and field veterinarians on effective parasite control management is a vital step in preventing AR. GIN control management should focus on accurate dosing, alternating anthelmintic classes, adjusting treatment frequency, and adopting new strategies such as TST combined with FEC. Given that the distribution of anthelmintic-resistant nematodes in Romania is largely unknown, establishing a national surveillance program for AR detection is essential. By providing farmers with the necessary information and resources, we can enhance the sustainability of parasite control and improve the overall health and productivity of goat herds.

## Figures and Tables

**Table 1:** Farming practices regarding parasitic infection control in 234 goat herds.

Farming Practice	Frequency or Manner of Performance	Number of Herds	Proportion of Herds [%]	95% Confidence Interval [%]
Number of dewormings per year	Never	3	1.3	0.4–3.7
	1 time	20	8.5	5.6–12.8
	2–3 times	149	63.7	57.3–69.6
	>3 times	62	26.5	21.3–32.5
Parasitological laboratory analyses (fecal egg count, FEC) per year	Never	207	88.5	83.7–92.0
	1 time	9	3.8	2.0–7.2
	2 times	10	4.3	2.3–7.7
	>3 or more times	0	0	0–1.6
	When clinical signs appear	8	3.4	1.7–6.6
	Always before treatment	0	0	0–1.6
The person responsible for deworming	The farmer	181	77.4	71.6–82.3
	Veterinarian	37	15.8	11.7–21.0
	Technician	16	6.8	4.3–10.8
	Others	0	0	0–1.6
Basis for selection of the time of treatment	Previous experience	165	70.5	64.4–76.0
	When clinical signs appear	27	11.5	8.1–16.3
	Before or after housing	20	8.5	5.6–12.8
	When changing pasture	12	5.1	3.0–8.8
	After parasitological analyses	8	3.4	1.7–6.6
	When weather changes	2	0.9	0.2–3.1
Manner of determining the dose	Visual approximation	191	81.6	76.2–86.1
	Weighing medium-sized animal	31	13.2	9.5–18.2
	Weighing the heaviest animal	6	2.6	1.2–5.5
	Individual weighting	6	2.6	1.2–5.5
	Other methods	0	0	0–1.6
Basis for choosing the antiparasitic product	Effectiveness	109	46.6	40.3–53.0
	Withdrawal time	63	26.9	21.7–33.0
	Habit	29	12.4	8.7–17.2
	Price	25	10.7	7.3–15.3
	Route of administration	8	3.4	1.7–6.6
	Parasitological laboratory analysis	0	0	0–1.6
	Other	0	0	0–1.6

**Table 2:** The farmers’ choice of anthelmintics against internal parasites (2021–2023) in 234 goat herds.

Anthelmintic Classes	Number of Herds	Proportion of Herds [%]	95% Confidence Interval [%]
Benzimidazoles (BZs)	200	85.5	80.4–89.4
Macrocyclic lactones (MLs)	191	81.6	76.2–86.1
Levamisole (LEV)	14	6.0	3.6–9.8
Other	2	0.9	0.2–3.1
Alternative treatments (e.g., medicinal plants)	6	2.6	1.2–5.5

**Table 3:** The prevalence of clinical signs considered by farmers to be associated with parasitic infections in 234 goat herds.

Clinical Symptoms	Number of Herds	Proportion of Herds [%]	95% Confidence Interval [%]
Cough	109	46.6	40.3–53.0
Diarrhea	103	44.0	37.8–50.4
Weakening	96	41.0	34.9–47.4
Submandibular edema	86	36.8	30.8–40.1
Decrease in milk yield	74	31.6	26.0–37.8
Reduced growth	72	30.8	25.2–37.0
Anemia	69	29.5	24.0–35.6
Sudden death	67	28.6	23.2–34.7

**Table 4:** Relationship between various characteristics of herds and the subjective role of parasites in the herd.

Factor ^a^	Herds in Which Parasites Were Considered a Problem (*n* = 42)	Herds in Which Parasites Were Not Considered a Problem (*n* = 192)	*p*-Value
Herd size ^b^	94, 70–173 (30–690)	150, 89–221 (12–718)	0.023
NUTS macro-region RO1	24/42 (57.1)	48/192 (25.0)	<0.001
Pasture			
Intensive farming system	0/42 (0)	18/192 (9.4)	0.049
Grazing in backyard ^c^	5/42 (11.9)	2/174 (1.2)	0.002
Grazing with cattle ^c^	13/42 (31.0)	64/174 (36.8)	0.475
Grazing with sheep ^c^	33/42 (78.6)	118/174 (67.8)	0.162
Grazing with horses ^c^	6/42 (14.3)	7/174 (4.0)	0.023
Grazing with roe deer ^c^	0/42 (0)	10/174 (5.8)	0.216
Grazing with buffalo ^c^	5/42 (11.9)	0/174 (0)	<0.001
Clinical symptoms			
Anemia	24/42 (57.1)	45/192 (23.4)	<0.001
Diarrhea	24/42 (57.1)	79/192 (41.2)	0.059
Submandibular edema	30/42 (71.4)	56/192 (29.2)	<0.001
Reduced growth	30/42 (71.4)	42/192 (21.9)	<0.001
Cough	36/42 (85.7)	73/192 (38.0)	<0.001
Decreased milk yield	28/42 (66.7)	46/192 (24.0)	<0.001
Weakening	31/42 (73.8)	65/192 (33.9)	<0.001
Sudden death	21/42 (50.0)	46/192 (24.0)	0.001
Internet as a source of knowledge	12/42 (28.6)	19/192 (9.9)	0.003

^a^ Presented as count and proportion [%] unless otherwise stated; ^b^ presented as a median, interquartile range, and range in parentheses; ^c^ calculated only for herds in which an extensive farming system was practiced.

## Data Availability

The data used in this study are available from the corresponding author upon reasonable request.

## Supplementary Materials

The following supporting information can be downloaded at: https://www.mdpi.com/article/10.3390/ani14162375/s1, Questionnaire regarding gastrointestinal parasites in dairy goat herds in Romania.
