# Supplementary material for: An Insight into Practices Associated with the Control of Internal Parasites in the Dairy Goat Herds of Romania: A Questionnaire Survey
Source: Animals (Basel). 2024 Aug 16;14(16):2375. doi: 10.3390/ani14162375 (PMC11350891; doi:10.3390/ani14162375)
Supplement: Supplementary file 1 [file animals-14-02375-s001.zip › animals-3146144-supplementary.pdf]

Questionnaire regarding gastrointestinal parasites in dairy goat herds in Romania

Dear goat farmer!

This questionnaire is part of a bigger project to investigate the gastrointestinal parasites in goats in Romania. The study will be conducted by USAMV Cluj-Napoca in collaboration with CAPRIROM Goat Association. The questionnaire should take less than 10 minutes to complete.

Gastrointestinal nematodes (GINs) are widespread and are an important cause of clinical disease and production losses in goats. The background of this project is that a lack of effect (resistance) has been found for drugs used to treat and prevent gastro-intestinal parasites in goats in Romania. This leads to challenges related to the practical handling of infection, especially regarding the nematode *Haemonchus contortus.* Therefore, we want to investigate the prevalence of different parasites, theeffects of treatment, and the consequences they may have on production.

Based on the information obtained from this questionnaire, we would like to invite some farmers to participate in a sampling program during the summer of 2024/2025 to assess the efficacy of anthelmintics. Fecal samples from goats will be collected from different parts of the country. This is a good opportunity to check the parasite-burden and the effect of anthelmintic treatment in your own flock. The data will not be anonymous to us as we will see the questionnaire results, but all answers will be handled confidentially, and all results will be anonymized when published.

Thank you for your participation!
Best regards,
Adrian-Valentin Potârniche
Lecturer Dr., Resident ECSRHM

Farm ID*:
Region*:
County*:

I. INFORMATION ON THE HOLDING

1) Which breed(s) of goats do you keep?

a) Carpathian
b) French Alpine
c) White of Banat
d) Saanen
e) Anglo-Nubian
f) Murcia
g) Mixed between : ____
h) Other: ____

2) Specify the herd-size (adult goats):

____

3) Do you have other livestock at your farm?

a) Cattle
b) Horses
c) Sheep
d) Other: ____
e) No

4) What type of flooring do your goats have during winter/housing period?

a) Slatted floor (expanded metal, plastic, wood, composite)
b) Solid floor with straw/wood shavings
c) Both solid and slatted floor

5) What kind of system?

a) Extensive
b) Intensive
c) Mixt

6) In which month were the goats turned out onto pasture?

a) March

b) April
c) May
d) June
e) Never
f) All year round
g) Other (specify): ____

7) In which month were the goats housed in the winter/autumn?

a) October
b) November
c) December
d) January
e) Never
f) On pasture all year round
g) Other (specify):____

8) What kind of pasture do the goats use during the different seasons?

a) Normal pasture
b) Cultivated pasture
c) Home pasture (backyard)
d) Rangeland
e) Forest pasture
f) Other (Specify):____

9) What kind of water source do the animals have access to on pasture?

a) Surface water (e.g. natural ponds, streams, lakes)
b) Trough with surface water
c) Trough with treated water

10) Which other species use the same pasture together with your goats (mixed grazing)?

a) No mixed grazing
b) Sheep
c) Cattle
d) Wild deer
e) Other (specify):____

II. INFORMATION REGARDING PARASITE CONTROL PRACTICES

11) Are newly purchased animals treated with anthelmintics before being introduced to your herd?

Before purchase:

a) Yes
b) No
c) Unknown

After purchase:

a) Yes
b) No
c) Unknown

12) Is any form of quarantine (housed/kept separately etc.) used in conjunction with purchase of animals?

a) Yes
b) No
c) Unknown

13) How often are you in contact with the veterinarian regarding parasite control in one year?

a) 0 (never)
b) 1-2 (rarely)
c) 3 or more (often)
d) always

14) How often are parasitological analyses performed in your herd (FEC)?

Adults

a) Never
b) once a year
c) twice a year
d) Before deworming
e) On suspicion: (diarrhea, other symptoms)

15) Who do you consider to be your most important advisor regarding treatment against parasites, graded from 1 to 5, where 1 is the most important and 5 is the least important advisor?

a) Internet
b) Veterinarian
c) Farmers community

d) Pharmacist
e) Neighbors, Colleagues, family
f) Other (specify):____

16) The most important decision-making factor when using an anthelmintic product?
a) Price
b) Withdrawn time
c) Effectiveness
d) Previous experience
e) Parasitological analyses
f) Way of administration
g) Others

17) Who is making the deworming of the goats in the herd?

a) Me (the owner)
b) The vet
c) Me, and occasionally the vet
d) Other: ________________________________

18) From where are you buying the deworming products?

a) From veterinary pharmacy
b) From veterinary personnel
c) From other farmers/friends
d) Other: ____

19) Before starting a deworming treatment you are speaking with a vet about it?

a) Yes
b) No
c) Sometimes

20) How do you decide the timing of treatment against parasites in your herd?

a) Experience from previous years
b) Weather and climate
c) Parasitological analysis
d) Pasture Rotation
e) Regularity, ex. every 3 months
f) Disease (death/diarrhea/reduced weight gain)
g) Housing for the winter
h) Other, specify: ____

21) How do you estimate the dose?

a) Individual weighing
b) Visual appraisal
c) Weigh medium-sized animal, and dose the rest based on this
d) Weigh heaviest animal, and dose the rest based on this
e) Other, specify: ____

22) What are you using to give the oral products?

a) Seringe
b) Drench gun
c) I put them in the water/food
d) Other (specify):______________________________

23) Have you known that the dose for goats in almost all deworming products is double than in sheep and cattle?

a) Yes
b) No

24) Are you satisfied by the guidance you receive from the local vet?

a) Yes
b) No
c) I will not answer

III. ANTHELMINTIC TREATMENT

25) How often do you treat against internal parasites? (especially GINs)

a) Never
b) Once a year
c) 2-3 times a year
d) More often

26) Why do you treat against internal parasites?
a) Prophylactic – I haven't had problems before

b) Prophylactic – I have had problems before
c) Treat when symptoms/disease observed
d) Not applicable

27) Which anthelmintic preparation (s) have you used in the following years? (check the box)

	2021	2022	2023
Benzimidazoles			
Macrocyclic lactones (avermectine)			
Levamisole			
Other			

28) Have you ever used alternative treatments for internal parasites?

a) Yes
b) No

IV. FARMERS' PERCEPTION OF PARASITES

29) Have you seen any of the following in your herd during the last 3 years? (check a box)

	Yes	No	Unknown
Pale/anemia			
Cough			
Diarrhea			
Weakening			
Submandibular oedema			
Reduced growth			
Sudden death			
Decrease in milk production			

30) Which parasites do you have in your herd? (check the box)

	Present	Not present	Unknown
GINs			
Tapeworms			

Liver flukes			
Lungworms			
Coccidia			
External parasites			

31) Do you think the treatment were effective?

a) Yes

b) No

32) Can you change the pasture from one year to another?

a) Yes

b) No

33) Did you know that changing the pasture can reduce parasitic infections?

a) Yes

b) No

34) Have parasites in goats been an increasing problem in your herd?

a) Yes

b) No
